# A study comparing dusting to basketing for renal stones ≤ 2 cm during flexible ureteroscopy

**DOI:** 10.1590/S1677-5538.IBJU.2022.0382

**Published:** 2022-12-20

**Authors:** NaiKai Liao, ShuTing Tan, ShuBo Yang, GaoQiang Zhai, ChengYang Li, TianYu Li, Yang Chen, LinJian Mo, JiWen Cheng

**Affiliations:** 1 Department of urology first affiliated hospital Guangxi medical university Nanning Guangxi China Department of urology, the first affiliated hospital of Guangxi medical university, Nanning, Guangxi, China

**Keywords:** Kidney Calculi, Ureteroscopy, Minimally Invasive Surgical Procedures

## Abstract

**Objectives:**

To compare the dusting efficiency and safety with basketing for treating renal stones ≤ 2 cm during flexible ureteroscopy (fURS).

**Materials and methods:**

This study included 218 patients with renal stones ≤ 2 cm treated with fURS. Among them, 106 patients underwent dusting, and 112 patients underwent fragmentation with basket extraction. All patients were followed up for 3 months postoperatively. The operating time, lasing time, stone-free rate (SFR) and complication rate were compared.

**Results:**

The mean stone size in the dusting group was 1.3 cm, whereas 1.4 cm in the basketing group. The mean operative time was significantly lower in the dusting group than in the basketing group (43.1±11.7 minutes VS 60.5±13.4 minutes, P <0.05), but the lasing time was significantly longer for the dusting group than for the basketing group (17.7±3.9 minutes VS 14.1±3.6 minutes, P <0.05). SFR was signiﬁcantly higher in the basketing group immediately after the operation and follow-up after 1 month (76.8% vs 55.7%, P= 0.001 and 88.4% vs 78.3%, P = 0.045). However, the SFR was similar for both groups (88.8% in the dusting group vs. 90.2% in the basketing group) after 3 months postoperatively. There was no statistical difference in the complication rates between the two groups.

**Conclusions:**

Dusting has advantages in shortening the operation time and reducing the operation cost, but the lasing time was longer compared with the basketing. Although there is no difference in long-term effect, basketing is superior to dusting in terms of short-term SFR. Moreover, dusting should be avoided in some special cases and basketing a better choice. Both techniques are effective for the treatment of renal stones ≤ 2 cm and choice depends on patient demographic and stone characteristics.

## INTRODUCTION

Many treatment options are available for patients with renal stones ( 1 ). Flexible ureteroscopy (fURS), characterized by minimally invasive and fast recovery, is more effective than SWL and safer than PCNL ( 2 ). It is currently recommended by the European Association of Urology (EAU) as one of the best choices for renal stones ≤ 2 cm ( 3 ). With the innovation of technology, fURS are now widely used in treating renal stones ≤2 cm ( 1 , 4 ). It can also be considered for stones > 2 cm, especially for patients who are poor candidates for PCNL due to anatomic challenges, medical comorbidities, and an inability to stop anticoagulation ( 5 , 6 ).

The main concerns of fURS are how to achieve optimal stone clearance with a minimal rate of complications. One of the techniques which has been described as ‘basketing’, is using a basket for active extraction of fragments after the primary stone has been broken into 3-4 mm size ( 7 ). The other option has been described as ‘dusting’. This technique uses laser to disintegrate the stone into tiny dust-like particles (mostly mentioned ≤ 2 mm), which can pass spontaneously through the ureter ( 8 , 9 ). There are limited clinical trials comparing dusting and basketing published ( 7 - 9 ). However, studies comparing these two techniques in a large sample size have not been published yet. There is currently no evidence to prove which technique is better. What are the advantages of dusting compared with basketing and will dusting become a better choice for the treatment of renal stones? The present study was conducted to address these issues to compare the clinical results of dusting and basketing during fURS.

## PATIENTS AND METHODS

### Patients

Our study included 218 patients with renal stones ≤ 2 cm treated with fURS (dusting or basketing) using a 100W high-power Ho: YAG system in our department from March 2018 to January 2021. All patients were prospectively randomized into two groups and informed consent (IRB number: NO.2018-KY-E-276) prior to the procedure. Patients who could not complete the procedure due to ureteral stenosis or anesthesia problems were excluded. Abdominopelvic computed tomography (CT) was done to determine the stone size and location, with CT density value. Patient demographics, routine serum creatinine examination outcomes, urine analysis and culture were also prospectively recorded ( Table-1 ). Patients were treated with a single dose of third-generation cephalosporin before the operation. Patients with positive urine cultures received culture-specific antibiotics until the urine culture results were negative before any intervention.

**Table 1:** Patient baseline characteristics in the Dusting versus Basketing Cohorts.

	Dusting (N=106)	Basketing (N=112)	P
Age (years,Mean±SD)	46.6±12.7	47.2±13.2	0.748#
Gender (male, %)	67(63%)	65(58%)	0.435*
**Side, n (%)**			0.301*
Right	40 (37.7)	50 (44.6)	
Left	66 (62.3)	62 (55.4)	
**Stone number, n (%)**			0.593*
Single	52 (49.1)	59 (52.7)	
Multiple	54 (50.9)	53 (47.3)	
**Stone location, n (%)**			0.632*
Renal pelvis	24 (22.6)	30 (26.8)	
calyx	53 (50.0)	49 (43.7)	
Multiple sites	29 (27.4)	33 (29.5)	
Stone size (mm, Mean±SD)	13.5±3.8	14.3±3.7	0.099*
UTI (positive culture)	16 (15.1)	18 (16.1)	0.842*
Creatinine (µmol/L, Mean±SD)	86.2±20.1	88.7±21.4	0.373#

*Results assessed statistically using the chi-squared test; #Results assessed statistically using the Student t test.

### Surgical procedures

All patients received general anesthesia and were operated on by the same surgeon in the lithotomy position. We first used the standard ureteroscopy technique to insert a guide wire into the renal pelvis. Then a ureteral access sheath (UAS) (Proxis, Boston Scientific, MA, United States) was placed through the guide wire for basketing patients and optional for dusting patients (patients with tortuous ureters or to obtain a fragment for analysis). The size of the UAS was 12/14Fr. The tip of the UAS was placed in the ureteropelvic junction. Afterward, a flexible ureteroscope (Olympus, Japan) was inserted to observe the pelvicalyceal system’s structure and identify the stones’ location.

A 100W high-power Ho:YAG system and 200 μm reusable laser fibers were used for lithotripsy irrespective of the stone size or location (Lumenis, Inc.). We renewed the tip of laser fibers using simple sterile scissors before every operation. The pulse energy settings used were 0.2–0.4 J with a frequency of 30–60 Hz giving a total power of 6–24 W in the dusting group. We gently placed the tip of the laser fiber over the stone surface and dusted stones into tiny pieces (≤ 2 mm) which can pass spontaneously. While for the basketing group, the procedure was completed using the energy of 0.8–1.2 J and a frequency of 8–10 Hz giving a total power of 8–12W.The stones were broken into 2–4 mm fragments that can be actively extracted using a nitinol basket rather than leaving it in situ for spontaneous passage. After completing the lithotripsy, the degree of injury to the ureter caused by the UAS was evaluated during the withdrawal of the fURS. Finally, a 4.7 F ureteric stent was placed at the end of the procedure.

### Follow-up

After the operation was completed, the operative time was recorded. On the first day after surgery, all patients were requested to have plain abdominal radiography (KUB) to confirm the proper placement of the double-J stents. The patients were then discharged within 24 h after surgery and received alpha-blocker therapy (tamsulosin 0.4mg daily) for 1 month. However, patients with complications such as fever were discharged after treatment. KUB was again performed after 4th week; if the patient had no kidney stone in the report, an ultrasound was also recommended to double-check the SFR. The stents were removed for those patients without residual fragments, or the residual fragments were smaller than 4mm. If the patients had residual stones bigger than 4mm or too many stone fragments which would not pass spontaneously after 4 weeks, we removed the double J after 6 weeks postoperatively. Whilst if the patients had residual stones which required a second session of fURS, the stents would be removed in the operating theatre. KUB and renal ultrasonography were again performed to reevaluate SFR after 3 months postoperatively. SFR was defined as no residual fragments of any size on KUB and renal ultrasonography. Postoperative complications were classified using the modified Clavien classification.

### Statistical analysis

The continuous variables were compared using means value (SD) with the Student t test, while the chi-squared test was used for categorical variables. Statistical analyses were performed with the Statistical Package for the Social Sciences (SPSS), version 18.0 for Windows. A p<0.05 was considered statistically significant for all analyses.

## RESULTS

The study included a total of 218 consecutive patients. Among them, 106 patients underwent ‘dusting’ and 112 underwent ‘basketing’. The mean stone size in the dusting group was 1.3 cm (0.5–1.9 cm) and 1.4 cm (0.7–2.0 cm) in the basketing group. There was no statistically significant difference in patients’ baseline demographic characteristics between the two groups ( Table-1 ).

The operation data and postoperative outcomes are presented in Table-2 . The mean operative time was significantly lower in the dusting group than in the basketing group (43.1±11.7 minutes vs 60.5±13.4 minutes, p <0.05), but the lasing time was significantly longer for the dusting group than for the basketing group (17.7±3.9 minutes vs 14.1±3.6 seconds, p<0.05). Both the groups had similar overall complication rates and the total period of hospital stay. Ureteric perforation (Grade 3 injury) occurred in 1 patient in the basketing group, which took place during the removal of the UAS and was treated by placing ureteric stents for 4 weeks. No gross hematuria was encountered in the groups. Postoperative fever (> 38°C) was seen in 4 patients in the dusting group, whereas 3 patients in the basketing group and were successfully treated by antibiotics therapy. One patient in the dusting group was admitted to the intensive care unit (ICU) due to septic shock and was successfully treated with culture-specific antibiotics.

**Table 2:** Operative and Follow-up outcomes between the Dusting and Basketing cohorts.

	Dusting (N=106)	Basketing (N=112)	P
Postoperative creatinine (µmol/L,Mean±SD)	89.9±16.8	90.5±17.0	0.805^#^
Access sheath used, n (%)	23(21.7%)	112(100%)	<0.05^*^
Operative time(min,Mean±SD)	43.1±11.7	60.5±13.4	<0.05^#^
laser time(min,Mean±SD)	17.7±3.9	14.1±3.6	<0.05^#^
Hospitalstay (days,Mean±SD)	1.2±0.5	1.3±0.6	0.673^#^
**Complications, n (%)**			**0.563** ^*^
Intraoperative	0(0%)	1(0.9%)	
Postoperative	4(3.8%)	3(2.7%)	
Symptoms due to fragments	16(15.1%)	13(11.6%)	0.550 ^*^
Second session of fURS	10(9.4%)	8(7.1%)	0.539 ^*^
**Stone-free rate, n (%)**			
1 day PO	59 (55.7%)	86 (76.8%)	0.001 ^*^
1 month PO	83(78.3%)	99 (88.4%)	0.045 ^*^
3 months PO	94 (88.8%)	101 (90.2%)	0.719 ^*^

*Results assessed statistically using the chi-squared test; #Results assessed statistically using the Student t test.

The immediate SFR after surgery was significantly higher in the basketing group (76.8%) compared with the dusting group (55.7%, p=0.001). The SFR was also higher in the basketing group at 88.4 % vs. 78.3% (p=0.045) than in the dusting group after 1 month postoperatively. However, the SFR was higher and similar for both groups (88.8% in the dusting group vs 90.2% in basketing group, P=0.719) during the follow-up period after 3 months postoperatively. The secondary session of fURS was required in the dusting group and basketing groups, in 9.4% and 7.1% (P=0.539) of patients, respectively. There was no statistical difference in postoperative creatinine and symptomatic residual fragments between the two groups.

## DISCUSSION

In recent years, fURS have become the most common treatment for renal stones ≤ 2 cm due to its minimally invasive characteristic and short learning curve ( 10 - 12 ). There are two alternative strategies for fURS. The first is fragmenting the stone, then basketing of fragment, and the second is stone dusting followed by spontaneous passage. Basketing uses high power and low frequency to break the stones into 2 to 4 mm fragments, followed by active removal with a basket through the UAS until all visible fragments have been cleared. This technique theoretically provides for a complete stone removal rate under direct visualization. A stone sample is also available for analysis, which will help provide accurate metabolic therapeutic treatment and lifestyle modification. Many clinical studies have demonstrated its safety and efficacy for many years ( 7 , 9 , 13 , 14 ). However, the high cost is frequently cited as the main drawback of this technique, as active extraction generally has longer operative times and requires a disposable basket and a UAS ( 15 , 16 ). Unlike basketing, the presence of a dusting technique may offer an excellent solution to this problem by using low power and high frequency to fragment stones into dust-like particles for spontaneous passage rather than using the basket and possibly a UAS ( 8 , 17 ). Additionally, this procedure can eliminate the need for additional staff, as the surgeon can perform the procedure without much assistance. Moreover, dusting has been associated with shorter operating times reported by some authors, which can also reduce operating costs. However, according to some of the comparative studies, SFRs were similar between these two techniques ( 16 , 18 ). In our research, UAS usage rates were lower with dusting, and the immediate procedure cost was significantly reduced compared to basketing. Consistent with previous studies, the dusting group’s mean operative time was significantly shorter. Meanwhile, there were no statistical differences in SFR and complication rate after 3 months postoperatively follow-up. As mentioned above, dusting appears to be the better choice for the fURS.

However, the potential risk factors, such as recurrent stone formation due to dust failing to pass, were also described in some studies, especially in patients with lower pole stones or acute infundibulo-pelvic angle ( 16 , 19 ). Lower pole stone is a challenging clinical entity and account for approximately 35% of renal stones. The lower pole stone with an acute infundibulo-pelvic angle not only increases the technical difficulty which needs better surgeon skill and experience, but also relates to the fragment clearance after operation due to the anatomy ( 19 , 20 ). This may be a disadvantage of the dusting technology, which needs further research in the future. In our study, we found that stones encrusted with abscess substance were also difficult to pass. The possible reason was that the small stone fragments will soon be covered by abscess substance after the stones are dusted into fragments, which will make it fail to pass. On the other hand, the dusting technique fragments stones into dust, resulting in the frequent poor vision field, especially for larger stones, and makes it difficult for surgeons to ensure that the stone is dusted small enough to pass spontaneously. In this situation, surgeons normally increase irrigation flow rates in order to get a better field view, which may increase intra-renal pressure and rise potential complications, especially sepsis risk ( 21 ). Furthermore, our results showed that basketing could not give an advantage in the complete SFR, while the immediate postoperative SFR and SFR observed 1 month postoperatively were significantly better in the basketing group. This may not increase short-term complications but influences treatment confidence and increases patient concern about the risk for long-term treatment due to repeated sessions of the same intervention, resulting in time lost for the patient. Moreover, working without active fragment retrieval and UAS can be associated with a shorter operating time in the dusting group, but this can lead to increased intraoperative pelvic pressure and the risk of postoperative infection. This was the most probable reason for the postoperative fever, which was more found in the dusting group than in the basketing group in our study. Among the total patient, only one of them developed septic shock, which was recorded in the dusting group.

When discussing the safety of both techniques, basketing theoretically increases the risk of injury because of the 100% use of UAS in these patients ( 22 , 23 ). However, this was not observed in our study. We did have one ureteric perforation in the basketing group, there was no statistical difference in complication rates between the two groups. In contrast, although the overall operative time was shorter in the dusting group, but the lasing time was significantly longer for the dusting group, especially in tackling the hard stones (CT value >800). Additionally, the power settings were also routinely higher during dusting. Studies showed laser has thermal effects on surrounding tissue and intra-renal temperature can reach 60ºC after only 10 seconds with 40W laser activation. Although the changes in temperature inside the renal pelvis during the procedure could not be confirmed in our study, longer lasing time might theoretically increase the risk of thermal damage to the renal collecting system ( 21 ). Aldoukhi and his colleagues evaluated the temperature change according to the fluid irrigation rate in a in vivo study. They reported that the internal temperature could be maintained under 50ºC with 40W laser activation when the irrigation flow rate was 40 mL/min. However, the temperature could be increased up to 70ºC when the irrigation flow rate was 15 mL/min ( 24 ). According to literature reports, tissue damage and cellular death will occur after short exposure of temperature above 40-60ºC. Temperatures above 43ºC could promote the protein denaturation of urothelium and therefore should be avoided ( 25 , 26 ). A longer follow-up study might be necessary to compare the ureteral stenosis between the two groups.

As previously stated, each method does have its own advantages and disadvantages. Thus, the question regarding which technique is better for treating renal stones remains controversial. An optimal approach should depend on the patient’s anatomic features and numerous stone factors, such as location, size, and density, as well as the patient’s economic conditions and personalized care. Therefore, it is clear that not all stones are suitable for a single approach. However, in our experience, dusting should be avoided in some patients: ( 1 ) patients having an acute infundibulo-pelvic angle with a long lower calyx or severe hydronephrosis, the stone fragments are easy to deposit in the lower calyx and are challenging to pass, resulting in residual stones. ( 2 ) the stones covered with abscess substance should be broken and retrieved by baskets as much as possible. ( 3 ) The hard stones with CT value >1000 need longer lasing time and higher power settings to slowly ablate the stone, which will increase the risk of thermal damage. Additionally, the damage to the pelvis mucosa will increase the viscosity of mucosa to stones, resulting in the stone fragments being difficult to pass spontaneously.

There are some limitations in our present study. Firstly, stone composition analysis has not been performed, which might explain its impact on lasing time. Secondly, patients used KUB and renal ultrasonography for the determination of SFR rather than CT, which may result in some detection bias in the SFR. Thirdly, our study did not present temperature changes inside the renal pelvis during surgery. Therefore, the thermal damage coming from longer lasing time or higher power settings was difficult to assess. Furthermore, the long-term differences over 6 months period results from the thermal injury were also unknown.

## CONCLUSIONS

Dusting has advantages in shortening the operation time and reducing the operation costs, but the lasing time was longer compared with the basketing. Although there is no difference in long-term effect, basketing is superior to dusting in terms of short-term SFR. Moreover, dusting should be avoided in some special cases and basketing may be a better choice. Both techniques have their relative advantages and disadvantages, they are all effective to treat renal stones ≤ 2 cm. The question regarding which technique is better depends on patient demographic and stone characteristics. However, future well-designed studies with longer follow-ups may be required to compare these two techniques for better results and improved recommendations.
